# Single Nucleotide Polymorphisms in* miR-122* Are Associated with the Risk of Hepatocellular Carcinoma in a Southern Chinese Population

**DOI:** 10.1155/2018/1540201

**Published:** 2018-12-19

**Authors:** Chunhua Bei, Shun Liu, Xiangyuan Yu, Moqin Qiu, Bo Tang, Weijia Liao, Songqing He, Hongping Yu

**Affiliations:** ^1^Department of Epidemiology and Health Statistics, School of Public Health, Guilin Medical University, 109 Huancheng North Road 2, Guilin 541004, China; ^2^Department of Epidemiology and Health Statistics, School of Public Health, Guangxi Medical University, 22 Shuangyong Road, Nanning 530021, China; ^3^Department of Hepatopancreatobiliary Surgery, The First Affiliated Hospital of Guangxi Medical University, Nanning, Guangxi, 530021, China; ^4^Laboratory of Hepatobiliary and Pancreatic Surgery, Affiliated Hospital of Guilin Medical University, 15 Lequn Road, Guilin 541001, China; ^5^Guangxi Key Laboratory of Molecular Medicine in Liver Injury and Repair, Guangxi, China; ^6^Department of Research, Affiliated Cancer Hospital of Guangxi Medical University, 71 Heti Road, Nanning 530021, China

## Abstract

Single nucleotide polymorphisms (SNPs) in microRNA may affect its expression and regulation of target genes, which may consequently alter individual susceptibility to cancer. In this study we aimed to investigate associations between* miR-122 *polymorphisms and hepatocellular carcinoma (HCC) in a southern Chinese population. Three selected SNPs in* miR-122 *(rs9966765, rs1135519, and rs17669) were genotyped in 1050 HCC patients and 1079 cancer-free controls using Sequenom MassARRAY platform and the associations of the three SNPs and HCC risk were evaluated. We found that individuals with the rs1135519 CC genotypes had a significant increased risk of HCC than those with TT genotypes (adjusted OR=2.71, 95% CI=1.15-6.36, and* P*=0.022), while the rs9966765 CC genotypes showed a borderline significant association with increased risk of HCC when compared with the GG genotypes (adjusted OR=2.38, 95% CI=0.99-5.75, and* P*=0.052). There was also a significant increased risk of HCC when combining risk genotypes of these loci, i.e., rs1135519 CC and rs9966765 CC. Compared with the low-risk group (0 risk genotype), the high risk group (1-2 risk genotypes) had significantly increased risk of HCC (OR=1.61, 95% CI=1.05-2.44, and* P*=0.028). Further genotype-expression analysis revealed that cases carrying the CC genotype of rs1135519 had lower levels of* miR-122* expression than those with the TT genotype. Our results suggest that SNP of rs1135519 modulates* miR-122* expression and contributes to the genetic susceptibility of HCC, either independently or together with rs9966765 in* miR-122.* Further well-designed studies with lager sample sizes are needed to confirm our findings.

## 1. Introduction

Hepatocellular carcinoma (HCC) is the second most common malignancies worldwide, with an estimated 782,500 new cases and 745,500 deaths reported annually, of which more than 50% are from China [1]. As the major risk factors, the relationships of hepatitis B/C virus (HBV/HCV) infection, tobacco use, and alcohol abuse and HCC have been well established [2, 3]. However, the exact mechanism of developing HCC is still unclearly. Recently, germline genetic variations, such as single nucleotide polymorphisms (SNPs), have been reported to be associated with HCC, which may help to uncover the molecular mechanism of development of HCC [4, 5].

MicroRNAs (miRNAs) are a class of small noncoding single-stranded RNAs that function as negative gene regulators by cleavage target mRNA or inhibiting its translation through binding to its 3'-untranslated region (UTR) [6, 7]. It is estimated that over 1,800 precursor miRNAs have been identified in the human genome, targeting 60% of the whole genes [8, 9]. MiRNAs are well conserved in eukaryotic organisms and considered to be a vital component of genetic regulation [10, 11]. Emerging studies have shown that miRNAs are involved in diverse biological processes, including development, differentiation, cell growth, and apoptosis [12]. Additionally, aberrant expression of miRNAs has been implicated in a wide variety of human cancers, including HCC [13].

*MiR-122 *is a highly abundant liver-specific miRNA that plays a pivotal role in liver development and hepatic function regulation [14, 15].* MiR-122* acts as a tumor suppressor against HCC by binding to the target genes involved in various biological processes in HCC, including cell proliferation, apoptosis, and angiogenesis [16, 17]. Recently, it has been also shown that downregulation of* miR-122* is correlated with metastasis and poor prognosis of HCC [18, 19]. Many studies have demonstrated that SNPs in miRNA can alter their expression level and may contribute to the susceptibility to cancer [20–22]. Given the important role of* miR-122* in liver pathology, we hypothesized that germline genetic variants within* miR-122 *would influence susceptibility to HCC. To test this hypothesis, we conducted a case-control study to investigate the association between SNPs in* miR-122* and the risk of HCC in a Chinese population.

## 2. Materials and Methods

### 2.1. Ethics Statement

This study was conducted in accordance with the approved guidelines by the Ethics Committee of the First Affiliated Hospital of Guangxi Medical University, and informed consent was obtained from all patients. And the Ethics Committee of the First Affiliated Hospital of Guilin Medical University approves that this study has complied with the Declaration of Helsinki.

### 2.2. Information and Sample Collection of Study Subjects

The present hospital-based case-control study included a total of 1050 HCC patients and 1079 cancer-free controls, which were consecutively recruited from First Affiliated Hospital of Guangxi Medical University and Affiliated Cancer Hospital of Guangxi Medical University from June 2007 to April 2011. Among the subjects, 589 HCC cases and 597 controls were collected between June 2007 and January 2010 as reported in our earlier studies [23]. Hence, an additional 461 HCC cases and 482 controls were recruited from February 2010 to April 2011. Altogether, a total of 1050 HCC cases and 1079 controls were used in the present study. All of the HCC patients were newly diagnosed and histologically confirmed. Those who had a prior history of other cancers, metastasized cancers, or previous radiotherapy or chemotherapy before the recruitment were excluded. The cancer-free controls were recruited from the Department of Orthopedics and Ophthalmology in the same period and were frequency matched to the cases by age (±5 years) and sex, without genetic relationships with the HCC patients. After signing an informed consent form, information including demographic data, history of tobacco and alcohol consumption and chronic HBV infection were obtained from each subject through face-to-face interviews conducted by trained investigators. Ever smokers were defined as persons who had smoked more than 100 cigarettes in their lifetime; ever drinkers were defined as persons who had used alcohol at least once a week for more than one year. HBV infection was defined as positive for HBV surface antigen (HBsAg). 5 mL of peripheral blood was collected for serology test and molecular genetic analysis. Thirty-two HCC cases with the CC or TT genotypes of rs1135519 were selected from cases with one of these two genotypes. Tumor tissues for these selected cases were collected from patients who had undergone surgery in the First Affiliated Hospital of Guilin Medical University.

### 2.3. SNPs Selection and Genotyping

The NCBI dbSNP database were used to selected common SNPs in 2kb upstream and downstream of* miR-122 *gene, with the minor allele frequency (MAF) > 0.05 in the CHB population (Chinese Han in Beijing). And the pairwise linkage disequilibrium (LD) had an r^2^ threshold of 0.8 on the NIEHS data-base (https://snpinfo.niehs.nih.gov/). Finally, rs9966765, rs1135519, and rs17669 in* miR-122* were selected for further study. DNA was extracted from peripheral blood by phenol–chloroform extraction and stored at -80°C. The three selected common SNPs in* miR-122 *were genotyped by using the Agena MassARRAY genotyping system (Agena, San Diego, CA) according to the manufacturer's instructions. Each PCR reaction mixture contained 10 ng of genomic DNA, 0.5 *μ*L 10×PCR Buffer, 0.4 *μ*L 25 mM MgCl _2_, 0.1 *μ*L25 mM dNTPs, 1 *μ*L0.5 uM primer Mix, and 0.2 *μ*L 5 U/*μ*L Hot Star Taq polymerase. Reaction was performed at 94°C for 15 min, followed by 45 cycles at 94°C for 20 s, 56°C for 30 s, and 72°C for 1 min, with a final incubation at 72°C for 3 min. The extension reactions were performed at 94°C for 30 s and then 94°C for 5 s, followed by 40 cycles at 52°C for 5 s, 5 cycles at 80°C for 5 s, with a final incubation at 72°C for 3min. Purified extension reaction products were spotted onto a 384-well Spectro CHIPs and measured by using the platform MALDI-TOF mass spectrometry within the Agena MassARRAY system. Genotype calling was performed and analyzed by using the MassARRAY Typer software version 4.0. All SNPs were successfully genotyped with high call rates (>94%). For quality control, we randomly selected 10% of the samples for repeating genotyping and found the consistent rate was 100%.

### 2.4. Quantitative Real-Time Reverse Transcriptase-Polymerase Chain Reaction Assay

*miR-122* expression was measured by reverse transcription-PCR according to the TaqMan microRNA Assay protocol (Bio Miao Biological Technology (Beijing) Co.,Ltd.). RNAs were extracted with TRIZOL reagent (Invitrogen, CA, USA) following the manufacturer's instructions were reverse-transcribed using a PrimeScript II 1st Strand cDNA Synthesis Kit ( TAKARA). The expression level of mature* miR-122 *was examined using SYBR FAST qPCR Kit Master Mix(2×) Universal (KAPA Biosystems), and the comparative C_t_ method comparing to the transcription level of U6 RNA was used to calculate the expression levels.

### 2.5. Statistical Analysis

The differences of distributions of selected variables and genotypes between cases and controls were assessed using *χ*^2^ test. Hardy-Weinberg equilibrium analysis was assessed by a goodness of fit *χ*^2^ test. The associations between* miR-122* SNPs and the risk of HCC were estimated by calculating the odds ratios (ORs) and 95% confidence intervals (95%CIs) with unconditional multivariate logistic regression model. All statistical tests were two-tailed. P values below 0.05 were regarded as indicating statistical significance. All analyses were performed using SPSS software, version 13.0 (SPSS Institute, Chicago, IL).

## 3. Result

### 3.1. Characteristics of the Study Population

The selected characteristics of 1050 HCC cases and 1079 cancer-free controls included in this study are shown in Table 1. There were no statistical differences in the distribution of age and sex between cases and controls (*P*=0.132 and 0.618, respectively). However, cases were more likely to be smokers (*P*<0.001), drinkers (*P*<0.001), and HBV carriers (*P*<0.001) than the controls.

### 3.2. Association between miR-122 Polymorphisms and the Risk of HCC

The primary information of the selected SNPs is listed in Table 2. The observed genotype frequencies for the SNPs of* miR-122* among the control subjects were all in agreement with HWE (*P*=0.544 for rs9966765,* P=*0.447 for rs1135519, and* P*=0.525 for rs17669, respectively). The calling rates were all above 94%. The distributions of genotype frequencies for these three SNPs in the cases and controls are summarized in Table 3. The frequencies of rs1135519 TT, TC, and CC genotypes were 68.65%, 28.37%, and 2.98% in HCC cases while they were 73.14%, 25.09%, and 1.77% in the controls, respectively. There were statistically significant difference in the distribution of rs1135519 genotypes between cases and controls (*P* =0.033). However, no significant difference in the genotype frequencies of rs9966765 or rs17669 was found (*P* = 0.071 for rs9966765 and* P* = 0.112 for rs17669, respectively).

After adjusting for age, sex, smoking, drinking, and HBV infection, we found that rs1135519 CC genotype was significantly associated with an increased risk of HCC when compared with TT genotype (adjusted OR=2.71, 95%CI=1.15-6.36, and* P*=0.022). We also found rs9966765 CC genotype had a borderline significant association with increased risk of HCC when compared with GG genotype (adjusted OR=2.38, 95% CI=0.99-5.75, and* P*=0.052). Although AG and GG genotypes of rs17669 tended to increased HCC risk (adjusted OR=1.17, 95% CI=0.96-1.42,* P*=0.123 for AG, OR=1.60, 95% CI =0.89-2.90, and* P*=0.117 for GG) when compared with AA genotype, however, it showed a nonsignificant association (Table 3).

### 3.3. Association Analysis of the Combined Risk Genotypes in miR-122 and HCC Risk

Considering a possible combined effect of the two SNPs, i.e., rs9966765 and rs1135519, on risk of HCC, we conducted combined analysis by using the number of the putative risk genotypes (i.e., rs9966765 CC and rs1135519 CC). As shown in Table 4, the combined risk genotypes were found to be associated with increased risk of HCC. The frequencies of 1-2 risk genotypes in HCC patients (2.9%) were higher than that in controls (1.8%), which showed statistical significance (*P*<0.05). Individuals with 1-2 risk genotypes had a significantly increased risk of HCC when compared with those harboring 0 risk genotypes after adjustment for age, sex, smoking, drinking, and HBV infection (adjusted OR =1.61, 95% CI=1.05-2.44, and* P=*0.028).

### 3.4. Correlation between rs1135519 Genotypes and the Expression Levels of miR-122

**Figure 1:**
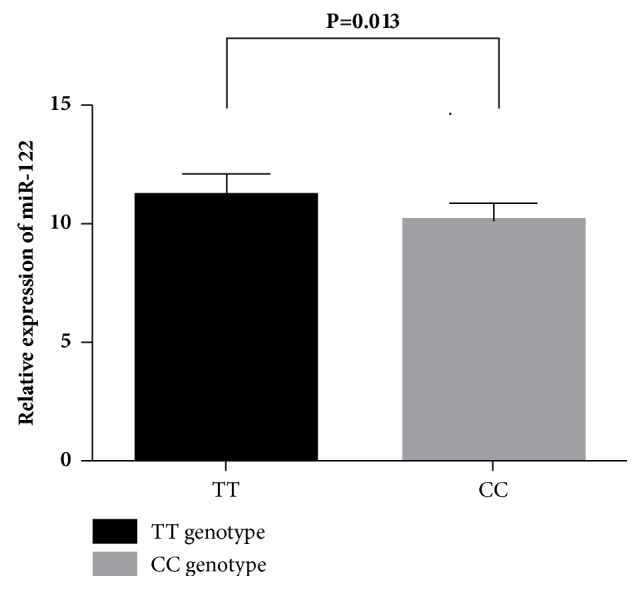
The relative expression of miR-122 in HCC tissues with rs1135519 CC and TT genotypes.* P* value is from* t* test.

To explore functional relevance of rs1135519 genotypes, we conducted genotype-phenotype correlation analysis between rs1135519 genotypes and* miR-122* expression level in 32 HCC tissues. Compared with individuals carrying TT genotype (n=28), rs1135519 CC genotype carriers (n=4) had lower levels of* miR-122* expression (∆Ct_mean_=10.13±0.75 for CC vs. ∆Ct_mean_=11.21±0.88 for TT,* P*=0.013, Figure 1), suggesting that rs1135519 SNP could modulate the* miR-122* expression.

## 4. Discussion

In this case-control study, we investigated the associations between three selected SNPs (i.e., rs9966765, rs1135519, and rs17669) in* miR-122* and risk of HCC by using 1050 HCC patients and 1079 cancer-free controls in a Chinese population. We found that polymorphisms of rs1135519 modulates* miR-122* expression and contributes to the genetic susceptibility of HCC, either independently or together with rs9966765 in* miR-122.*

As liver-specific miRNA,* miR-122* has been reported to be down-regulated and associated with development, metastasis, recurrence, and poor prognosis of HCC [18, 19, 24]. Reduced levels of* miR-122* in HCC may result in chromosomal instability through deregulation of cyclin G1 or p53-dependent pathways [25, 26]. Functional studies demonstrated that* miR-122* could suppress cell proliferation and induces cell apoptosis in HCC by directly targeting Wnt/beta-catenin pathway, while the activation of oncogene c-Myc may induce transcriptional repression of* miR-122* in HCC [27, 28]. Studies in mouse models using modified antisense* miR-122* showed that its depletion compromised the liver function and reduced cholesterol level by targeting expression of genes involved in cholesterol biosynthesis that was found to facilitate cystogenesis and hepatocarcinogenesis [29, 30]. Thus, it was speculated that special SNPs in* miR-122* gene might affect its expression and subsequently alter the risk of HCC. In this study, the CC genotype of rs1135519 was correlated with lower expression of* miR-122*, which was consistent with its risk effect on HCC.

Polymorphisms in miRNA with potential functions in its expression may influence the individual susceptibility to cancers, which have been well illustrated. Xu et al. [31] reported that HCC risk of rs2910164 GG genotype in* miR-146a* was 2-times of CC genotype among male (OR=2.02, 95% CI=1.06–3.85, and P=0.034). Further investigation disclosed that GG genotype conferred a higher expression level of mature* miR-146a*. Qi et al. [32] showed that the A to G base change of rs999885 in the promoter region of* miR-106b-25* cluster may provide an increased risk for HCC in HBV persistent carriers by altering the expression of the* miR-106b-25* cluster. Our previous research also showed that miR-199a rs74723057 polymorphism combined with MET rs1621 may influence susceptibility to HCC [33]. To date, only one published study had reported the effect of genetic variants in* miR-122* on risk of HCC. Liu et al. [34] found that the C to A base change of rs4309483 located in the regulatory regions of* miR-122 *was associated with decreased expression of* miR-122* and thus showed an increased risk of HCC in a case-control study, which was consistent with our study.

Several potential limitations in the current study should be considered. Firstly, as a hospital-based case-control study, the selection bias was unavoidable. To reduce the potential section bias, we had applied a rigorous design in selecting cases and controls by frequency matching with age and sex. Secondly, the sample size may limit the statistical power of our study, especially for subgroup analysis, although it had more than 2000. As a result, our results still need further validation. Finally, the precise molecular mechanisms underlying that how the SNP altering* miR-122* expression need to be illustrated in future work.

In conclusion, we identified two genetic variants in* miR-122* (rs9966765 and rs1135519), which may individually or jointly modulate the risk of HCC. Our results suggest the potential role the two SNPs in regulation of* miR-122* expression and development of HCC. Further larger and well-designed studies with diverse populations and functional assays are warranted to validate our findings.

## Figures and Tables

**Table 1:** Frequency distributions of the clinical features between HCC cases (n=1050) and controls (n=1079) [n(%)].

**Variables**	**Cases**	**Controls**	***P*** ^**a**^
**Age**			0.053
<48	463 (44.10)	521 (48.29)	
≥48	587 (49.00)	558 (51.71)	
**Sex**			0.618
Male	917 (87.33)	950 (88.04)	
Female	133 (12.67)	129 (11.96)	
**Smoking**			<0.001
Never	663 (63.14)	908 (84.15)	
Ever	387 (36.86)	171 (15.85)	
**Drinking **			<0.001
Never	698 (66.48)	937 (86.84)	
Ever	352 (33.52)	142 (13.16)	
**HBV infection**			<0.001
No	176 (16.76)	981 (90.92)	
Yes	874 (83.24)	98 (9.08)	

^a^ P-value for Chi-square test.

**Table 2:** The information of the miR-122 SNPs.

**SNP**	**Location**	**Base change**	**Genotypes**	**MAF ** ^**a**^	**P**_**HWE**_^**b**^	**Genotyping rate (**%**)**
**rs9966765**	5' near gene	G > C	GG CG CC	0.142	0.544	94.9
**rs1135519**	5' near gene	T > C	TT CT CC	0.144	0.447	94.9
**rs17669**	3' near gene	A > G	AA GA GG	0.143	0.525	94.6

^a^ Minor allele frequency;

^b^*P*-value for Hardy-Weinberg equilibrium tests.

**Table 3:** Association between SNPs in miR-122 and HCC risk [n (%)].

**Genotypes**	**Cases ** ^**a**^	**Controls ** ^**a**^	**OR (95**%** CI) **^**b**^	**P** ^**b**^
**rs9966765**				
GG	725 (69.38)	788 (73.58)	1	
CG	291 (27.85)	264 (24.65)	1.08 (0.80-1.46)	0.630
CC	29 (2.78)	19 (1.77)	2.38 (0.99-5.75)	0.052
**rs1135519**				
TT	714 (68.65)	786 (73.14)	1	
CT	295 (28.37)	270 (25.09)	1.09 (0.80-1.47)	0.563
CC	31 (2.98)	19 (1.77)	2.71 (1.15-6.36)	0.022
**rs17669**				
AA	725 (69.85)	789 (73.46)	1	
AG	285 (27.45)	266 (24.77)	1.17 (0.96-1.42)	0.123
GG	28 (2.70)	19 (1.77)	1.60 (0.89-2.90)	0.117

^a^ The numbers were not the same for each SNP due to different calling rates.

^b^ Adjusted for age, sex, smoking, drinking, and HBV infection in logistic regression model.

**Table 4:** Association of number of risk genotypes and HCC risk [n(%)].

**Number of risk genotype ** ^**a**^	**Case**	**Control**	**OR (95**%** CI) **^**b**^	**P** ^**b**^
0	1009 (97.1)	1050 (98.2)	1	
1-2	30 (2.9)	19 (1.8)	1.61 (1.05–2.44)	0.028

^a^ Risk genotypes used were rs9966765 CC and rs1135519 CC.

^b^ Adjusted for age, sex, smoking, drinking status and HBV infection.

## Data Availability

The data used to support the findings of this study are available from the corresponding author upon request.
